# Severe Babesiosis With Heavy Parasitemia in an Immunocompetent Patient Treated Successfully With Red Cell Exchange Transfusion

**DOI:** 10.7759/cureus.23344

**Published:** 2022-03-20

**Authors:** Raghavendra R Sanivarapu, Vaishali Kashyap, Javed Iqbal

**Affiliations:** 1 Pulmonary and Critical Care Medicine, Nassau University Medical Center, East Meadow, USA; 2 Medicine, Nassau University Medical Center, East Meadow, USA

**Keywords:** tick-borne infections, immuno-competent, parasitemia, exchange transfusion, severe babesiosis

## Abstract

Babesiosis is a zoonotic disease caused by a protozoa *Babesia*. It is transmitted through a tick vector and affects red blood cells. The clinical manifestation of Babesiosis varies and can range from asymptomatic infection to fatal multi-organ failure and death. The severity of infection varies with different *Babesia *species and also the immunity of the host. The management of Babesiosis depends on the severity of infection and the burden of parasitemia. Here we present a case of severe Babesiosis in an immunocompetent patient with heavy parasitemia requiring red blood cell exchange transfusion (ET).

## Introduction

Babesiosis is a parasitic infection caused by *Babesia* spp. The common species in the USA is *Babesia microti*. The geographical distribution of *Babesia* is similar to that of other tick-borne diseases, especially Lyme, and is mainly located in Northeastern and Midwestern parts of USA [1]. The common vector involved in the transmission of parasites is the nymphal stage of Ixodes scapularis. The severity of infection depends on the immune status of the host and also on different Babesia species and can range from asymptomatic infection to multi-organ failure and death [2-3]. Here we present a case of a young immunocompetent male presenting with high-grade unrelenting fevers from severe Babesiosis with multi-organ involvement requiring the use of red blood cell exchange transfusion (ET) to lower parasitic burden.

## Case presentation

A 46-year-old Hispanic male with no significant past medical history presented with the chief complaint of unrelenting fevers for two weeks associated with dull epigastric abdominal pain and watery diarrhea. He denied sick contacts, recent travel, and admitted to safe sex practices.

The patient’s vital signs at admission were blood pressure 125/62 mmHg, heart rate 120 bpm, respiratory rate 16 bpm, and he was febrile with a temperature of 102.7°F. His physical exam was significant for yellow conjunctiva, diaphoresis, with a tender epigastric region but otherwise soft and nondistended abdomen, no skin rashes, and no hepatomegaly could be discerned. He was admitted for sepsis of an unknown source and was started on IV fluids and broad-spectrum antibiotics with vancomycin and piperacillin-tazobactam.

Work up for sepsis was started and pertinent labs included normal white blood cell count of 7.12 K/mm3, hemoglobin of 6.7 g/dL, platelet of 85 K/mm3, serum creatinine of 2.1 mg/dL, alanine aminotransferase (ALT) 189 U/L, aspartate aminotransferase (AST) 389 U/L, alkaline phosphatase (ALP) 118 U/L, and lactate of 1.7 mmol/L. Other important labs included are reticulocyte count 19.70%, international normalized ratio (INR) of 1.1, prolonged partial thromboplastin time (PTT) 37.7 s, prolonged prothrombin time (PT) of 14.5 s, fibrinogen 847 mg/dL, and D-dimer of 2.74. Pertinent imaging included a chest X-ray with no consolidation or effusion; abdominal ultrasound showed only a 1.2-cm renal cyst and a CT abdomen/pelvis that showed no signs of bowel obstruction or other pathology.

Given the clinical history and labs, differential diagnoses considered at admission were sepsis secondary to intra-abdominal source, Thrombotic thrombocytopenic purpura/hemolytic uremic syndrome, malaria, hemorrhagic fever, Lyme disease, and *Babesia*.

**Figure 1:**
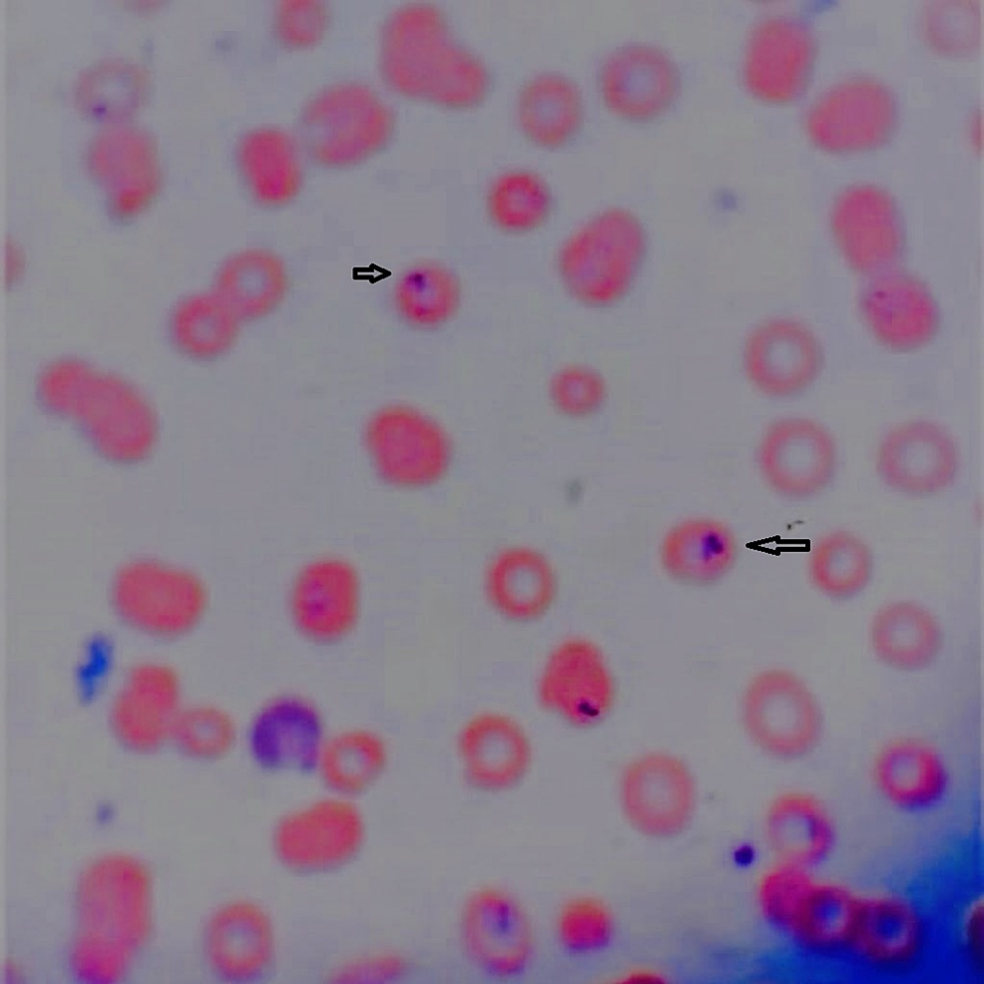
Peripheral blood smear of the patient showing intracellular inclusion bodies in red blood cells (arrows).

**Figure 2:**
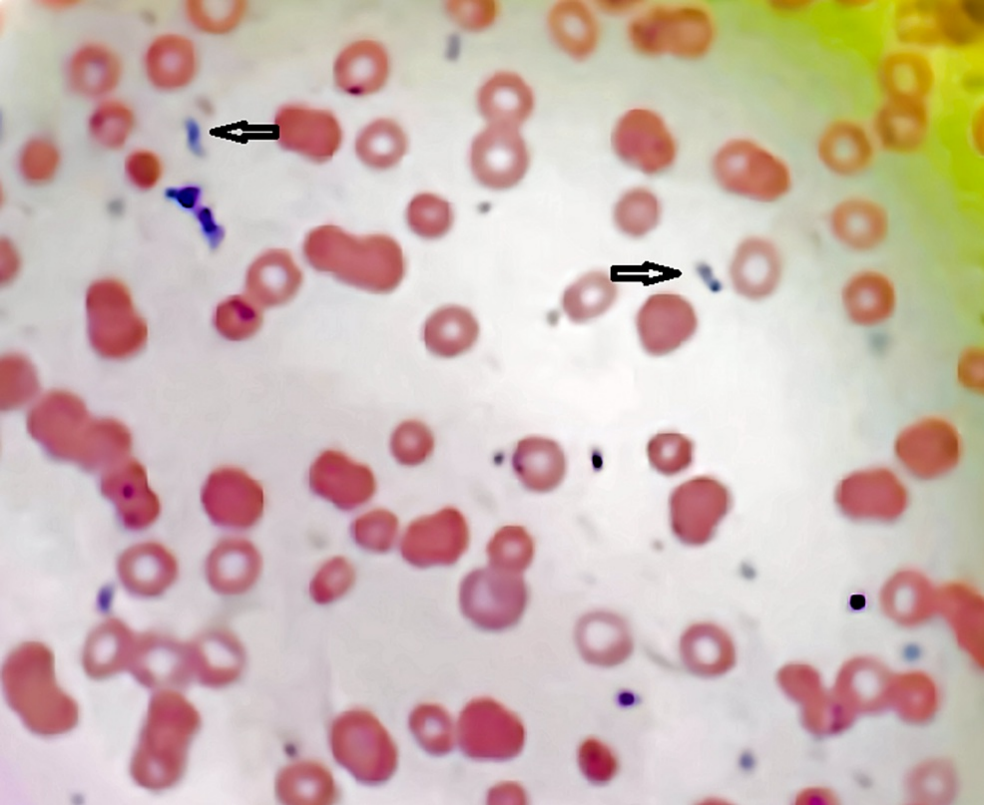
Peripheral blood smear of the patient showing extracellular inclusion bodies from Babesia spp. (arrows).

The patient clinically deteriorated and was noted to have respiratory distress and worsening thrombocytopenia and high-grade fever and he was moved to the intensive care unit (ICU). A peripheral smear was obtained and it showed both intra and extracellular inclusion bodies in red blood cells which was identified as *Babesia microti* (Figures 1-2). The smear did not show any schizocyte.

The patient’s antibiotics were changed to atovaquone, azithromycin, and clindamycin for severe Babesiosis. The patient’s parasite burden was noted as 15% and repeat labs showed continued hemolysis with worsening renal function and liver-related tests. Given severe Babesiosis a decision was made to perform red blood cell ET to lower the parasitic burden.

The patient received one cycle of red blood cell ET and his parasitemia reduced to 3.6% with improvement in all labs: anemia improved from 7.1 to 9.1 g/dL, creatinine improved from 1.9 to 1.7 mg/dL, liver-related enzymes improved as the following ALT from 172 to 116 U/L, AST from 361 to 258 U/L, ALP from 102 to 70 U/L, direct bilirubin improved from 4.1 to 1.9 mg/mL, LDH from 1508 to 1048 U/L, and fibrinogen from 847 to 571 mg/mL (Table 1). The patient became afebrile and improved clinically. The parasite count for the next three days continued to decrease to 1.2%, 0.3%, and then 0.05%. He was transferred to the regular floor and was discharged home eventually.

**Table 1:** Chart showing trending of labs pre and post red blood cell exchange transfusion. ALT, alanine aminotransferase; AST, aspartate aminotransferase; ALP, alkaline phosphatase

Lab parameter	Prior to exchange transfusion			Post exchange transfusion		
	On admission	Day 1	Day 2	Day 3	Day 4	Day 5
Hemoglobin (g/dL)	6.7	7.1	7.2	8.4	9.1	9.2
Platelets (K/mm^3^)	85	93	84	108	178	238
Serum creatinine (mg/dL)	2.1	1.9	1.8	1.7	1.0	0.9
ALT (U/L)	189	172	169	116	113	113
AST (U/L)	389	361	348	258	233	228
ALP (U/L)	118	102	102	83	78	70
Parasitemia (%)	15	15	15	3.6%	1.2	0.3

## Discussion

Babesiosis is a nationally notifiable tick-borne disease that can cause fatal multiorgan failure and death in an immunocompromised host. As per the Centers for Disease Control and Prevention (CDC) surveillance between the years, 2011 and 2015 of the total reported cases in the USA alone were 7,612 of which about 46% of patients require hospitalization [4]. A community-based serosurvey and case finding study conducted by Krause et al. showed that 20% of adults and 40% of children had an asymptomatic infection in endemic areas [5]. The risk factors for severe infection include immunosuppression from asplenia, HIV/AIDS with low CD4 count, chemotherapy, immunosuppressive therapy from a solid organ transplant, and age neonates or adults >50 years [5-7]. The common clinical symptoms include gradual onset fatigue, intermittent or sustained fevers, chills, and sweats. Less commonly, the patient can develop sore throat, abdominal pain, diarrhea, dark urine, shortness of breath, and weight loss [8]. The most common physical exam findings include fever and sometimes splenomegaly or hepatomegaly can be noted. Less common findings are icterus, jaundice, and retinopathy [9]. The mortality rate from severe infection is ~5% based on the retrospective study of 136 patients and even higher as ~21% in immunocompromised patients [10-11]. Other diseases that can mimic similar presentation include malaria, Lyme disease, viral hepatitis, and other tick-borne diseases. The diagnosis of Babesiosis is made by peripheral smear exam showing both intra and extracellular parasites and maltese cross formation or by polymerase chain reaction (PCR) test. 

The management of Babesiosis depends on the severity of symptoms and antibiotic therapy is reserved for symptomatic cases alone. The antimicrobials of choice are azithromycin and atovaquone or alternatively clindamycin with quinine is used [2]. The duration of therapy for azithromycin and atovaquone or alternate regimen is usually 7-10 days and can be prolonged for persistent parasitemia. Severe disease is considered when parasitemia is >4% or associated with severe symptoms including chills, fever, myalgia, nausea, vomiting, diarrhea, and in severe cases shortness of breath from acute respiratory distress syndrome can occur [9]. Splenic rupture is a rare complication and is seen in healthy young patients with low parasitemia [12].

The red blood cell ET as a treatment is rarely used except in severe Babesiosis patients with parasitemia >10% and associated end-organ dysfunction including severe hemolysis and severe pulmonary, renal, or hepatic compromise [2, 13]. The patient had severe Babesiosis and had a parasitic burden of 15% and had end-organ damage with raised liver-related tests and raised serum creatinine, and so ET was considered. The use of ET also removes pro-inflammatory cytokines and reduces the severity of infection. Thus far the use of ET for the treatment of severe Babesiosis is based only on a few case reports and no randomized control trial exists showing its efficacy. 

## Conclusions

In severe cases of Babesiosis with >10% parasitemia with end-organ damage such as liver and kidney failure, whole blood ET should be considered as a life-saving intervention along with antimicrobial therapy. Further studies are required to show the beneficial effect of ET in the management of severe Babesiosis.

## References

[1] Vannier E, Krause PJ (2012). Human babesiosis. N Engl J Med.

[2] Krause PJ, Auwaerter PG, Bannuru RR (2021). Clinical practice guidelines by the Infectious Diseases Society of America (IDSA): 2020 guideline on diagnosis and management of babesiosis. Clin Infect Dis.

[3] Wormser GP, Lombardo G, Silverblatt F (2011). Babesiosis as a cause of fever in patients undergoing a splenectomy. Am Surg.

[4] Gray EB, Herwaldt BL (2019). Babesiosis surveillance - United States, 2011-2015. MMWR Surveill Summ.

[5] Krause PJ, McKay K, Gadbaw J (2003). Increasing health burden of human babesiosis in endemic sites. Am J Trop Med Hyg.

[6] Hatcher JC, Greenberg PD, Antique J, Jimenez-Lucho VE (2001). Severe babesiosis in Long Island: review of 34 cases and their complications. Clin Infect Dis.

[7] Cullen G, Sands BE, Yajnik V (2010). Babesiosis in a patient on infliximab for Crohn's disease. Inflamm Bowel Dis.

[8] Vannier EG, Diuk-Wasser MA, Ben Mamoun C, Krause PJ (2015). Babesiosis. Infect Dis Clin North Am.

[9] Mareedu N, Schotthoefer AM, Tompkins J, Hall MC, Fritsche TR, Frost HM (2017). Risk factors for severe infection, hospitalization, and prolonged antimicrobial therapy in patients with babesiosis. Am J Trop Med Hyg.

[10] Meldrum SC, Birkhead GS, White DJ, Benach JL, Morse DL (1992). Human babesiosis in New York State: an epidemiological description of 136 cases. Clin Infect Dis.

[11] Krause PJ, Gewurz BE, Hill D (2008). Persistent and relapsing babesiosis in immunocompromised patients. Clin Infect Dis.

[12] Dumic I, Patel J, Hart M, Niendorf ER, Martin S, Ramanan P (2018). Splenic rupture as the first manifestation of babesia microti infection: report of a case and review of literature. Am J Case Rep.

[13] Spaete J, Patrozou E, Rich JD, Sweeney JD (2009). Red cell exchange transfusion for babesiosis in Rhode Island. J Clin Apher.

